# Prevalence of adenovirus respiratory tract and hiv co-infections in patients attending the University of Ilorin, teaching hospital, Ilorin, Nigeria

**DOI:** 10.1186/1756-0500-7-870

**Published:** 2014-12-03

**Authors:** Olatunji M Kolawole, Tolulope O Oladosu, Aishat A Abdulkarim, Anthony I Okoh

**Affiliations:** Department of Biochemistry and Microbiology, Applied and Environmental Microbiology Research Group, University of Fort Hare, Alice, South Africa; Department of Microbiology, Infectious Diseases and Environmental Health Research Group, University of Ilorin, Ilorin, PMB 1515 Nigeria; Paediatric Department, University of Ilorin Teaching Hospital, Ilorin, PMB 1459 Nigeria

**Keywords:** Adenovirus, HIV, Co-infections, ELISA, Ilorin, Nigeria

## Abstract

**Background:**

Adenovirus co-infections in HIV patients cause wide-spread morbidity in sub-Saharan Africa, but little research has documented the burden and distribution of these pathogens. This study was conducted between December, 2010 and March, 2011 to investigate the prevalence of Adenovirus Respiratory Tract and HIV co-infections in Patients attending the University of Ilorin Teaching Hospital Ilorin, Nigeria.

**Method:**

One Hundred and Eighty Four (184) patients were recruited with confirmed HIV positive status. Investigation was done by serology using the Human Adenovirus IgG ELISA Kit to test for the presence of the Immunoglobulin G (antibody) against the virus. This was conducted and juxtaposed simultaneously with responses received from the questionnaires provided to each participant to correlate the relationship of the co-infections to their socio-demographic factors (Age, Gender, Occupation and location of residence), risk factors (Average hours of exposure per day (time spent outdoor daily), proximity of their apartments to livestock settlements), recent occurrence of respiratory tract infections/conjunctivitis and their ART status.

**Results:**

This study recorded a prevalent rate of 38% (70 patients) to the co-infections. Nevertheless, 62% (114 patients) tested negative to the co-infections.

**Conclusion:**

There was statistical significance between the ages of HIV patients and Adenovirus co-infection (p < 0.05). However, there was no significance with respect to gender of the subjects (p > 0.05). The findings also showed that there were statistical significance for all the risk factors; Occupation, Location and Proximity to Livestock settlement, recent respiratory tract infection/conjunctivitis, and ART status in relation to Adenovirus and HIV co-infections (p < 0.05).

**Electronic supplementary material:**

The online version of this article (doi:10.1186/1756-0500-7-870) contains supplementary material, which is available to authorized users.

## Background

Adenoviruses belong to the family Adenoviridae which is a group 1 double-stranded DNA virus according to the Baltimore Classification Scheme of Viruses. A typical adenovirus measures 70-90nm possessing an icosahedral capsid [1]. It was first isolated in the 1950’s in adenoid tissue-derived cell cultures, giving rise to the name (Adenovirus). In humans, there are 56 accepted human adenovirus types (HAdV-1 to 56) in seven species (Human Adenovirus A to G) [2].

According to Ampel in 2008, in New Mexico; three cases of severe acute respiratory tract infection were reported, one of which resulted in death, these were associated with adenovirus 14 (Ad14). Another report by [3] tagged Ad14 serotype as an emerging cause of acute respiratory disease among US military recruits in 2005. Furthermore, from March through June 2007, One Hundred and Forty (140) additional cases were confirmed in the United States [3].

Unlike Adenovirus, HIV is a member of the genus Lentivirus part of the family of Retroviridae. Lentiviruses have many common morphologies and peculiar biological properties. HIV is different in structure from other retroviruses. It is roughly spherical with a diameter of about 120 nm, around 60 times smaller than a red blood cell [4]. HIV infection in humans is considered a pandemic by the World Health Organization (WHO). HIV progressively causes acquired immunodeficiency syndrome (AIDS) [5, 6]. This is a condition in humans in which the immune system falls, leading to life-threatening infections which are called opportunistic infections caused by other pathogenic organisms.

HIV was discovered in 1981 and till date, AIDS has killed more than 25 million people [7]. The virus infects about 0.6% of the world’s population. In 2005, AIDS claimed an estimated 2.4–3.3 million lives, of which more than 570,000 were children. About One third of these deaths occurred in Sub-Saharan Africa, where poor economic growth and poor standard of living was on the increase [8]. During this time, HIV was statistically predicted to infect 90 million people in Africa, which would result in a minimum estimate of 18 million orphans from this crisis [9]. Antiretroviral treatment reduces both the mortality and the morbidity of HIV infection. Routine access to antiretroviral medication in developing and under-developed countries which was unaffordable in the past has been aided by international Organizations.

Although, adenovirus infection is typically self-limiting, fatal disease may occur among persons infected with Human Immunodeficiency Virus (HIV) and other immunosuppressed individuals [10]. Among immunosuppressed persons, adenovirus infection is frequently associated with disseminated disease in which two or more organs are involved; viremia is frequently present [10].

Mortality for persons with disseminated adenovirus disease remains high despite the use of antiviral therapy [11]. Asymptomatic carriage of adenovirus is common in HIV-infected persons [12]; asymptomatic viruria occurs in up to 20% of persons with HIV/AIDS [13]; and asymptomatic adenoviremia has been reported in children who are HIV-seropositive [12].

Adenovirus infections occur in 12% to 28% of HIV-infected persons, and 45% of the infections result in death, typically within 2 months of diagnosis [14, 15]. Mortality is usually related to site of infection: and was noticed to be 60% in those with pneumonia and 50% in those with hepatitis [14]. Most patients with AIDS and adenovirus infection are co-infected with other pathogens or have other complicating conditions that may contribute to their mortality risk [16]. Adenovirus is used as a vehicle to administer targeted therapy in the form of recombinant DNA or protein [17].

According to [18] the average life expectancy of an HIV infected individual was observed to be 32 years from the time of infection if treatment is started when the CD4 count is 350/μL. Life expectancy is further enhanced if antiretroviral therapy is initiated before the CD4 count falls below 500/μL.

The impact of this study borders on the evaluation and reduction of adenovirus respiratory tract co-infections in HIV patients in the Ilorin Metropolis and its environs. Since there is no scientifically documented data on the epidemiology of the co-infections in Nigeria, it could serve as a reference point for the innovation and implementation of certain management schemes that will help better the lots of HIV/AIDS patients and the society at large.

## Methods

### Study site and design

This study was carried out at the Antiretroviral Therapy Clinic of the University of Ilorin Teaching Hospital, Ilorin, Kwara State, Nigeria. The facility is been supported by a non-indigenous, non-governmental organization – Institute of Human Virology, Nigeria (IHVN) in collaboration with the Hospital to provide adequate and intensive healthcare services to HIV positive patients both within the Ilorin metropolis and its environs. The research was conducted between December, 2010 to March 2011. The study was a cross-sectional descriptive research in which the individual’s current disease status was ascertained. Sample size of 184 patients recruited was determined by the Fisher’s method [19].

### Ethical clearance

An ethical clearance was obtained from the Medical Advisory Committee of the University of Ilorin Teaching Hospital for this research. The patients were adequately briefed about the purpose and benefits of the study and their consent was sought for before inclusion in the study. They were assured that any information arising from the study will be confidential. A follow – up counselling was provided to the patients after the test.

### Questionnaire

Instrument was administered through semi-structured close-ended questionnaires to determine the socio-demographic and risk factors associated with Adenovirus co-infections with HIV. Questions were translated into local languages or explained to the patients where necessary (Additional file 1).

### Enzyme linked immunosorbent assay (ELISA)

ELISA was done using the Human Adenovirus IgG (ADV-IgG) ELISA kit (Cusabio Biotech Co.Ltd, China). Those found positive were confirmed by rapid and simple assays [20].

### Sample collection

Blood samples, 3–5 ml were collected aseptically by ante-cubital venipuncture from patients, after obtaining pre-informed consent in the prescribed format. The sera samples collected after centrifugation at 2500 g were stored at −4°C until the assays were performed.

### Assay procedure

Sera samples were diluted and tested according to the Kit’s specification and manufacturer’s instructions.

### Data analysis

Statistical Package for Social Sciences Version 17.0 (SPSS Inc., Chicago, IL) was used for all analyses. Descriptive statistics such as Standard Deviation, Frequency counts and Percentages were used in the computing of the results in order to give a lucid representation of the data analysed. The Chi-square (X^2^) test was used to test for significant differences and effects at p < 0.05. Results were also presented in tables and charts.

## Results

The prevalence rate of Adenovirus co-infections with HIV in the patients was observed to be 66.7% in the age group of 5-12years and age group of 13-19years simultaneously having the highest prevalence rate. However, it was 30.8% in age group (28–39 years) being the group with the lowest prevalence rate. The overall total population of patients having Adenovirus co-infections with HIV was 70 accounting for 38% prevalence, while the rest (114) tested negative to Adenovirus while being positive for HIV infections (Table 1). There was statistical significance between the ages of HIV patients and Adenovirus co-infections.

**Table 1:** **Prevalence of adenovirus co-infections in HIV patients with respect to age, gender, recent occurrence of respiratory tract infections, conjunctivitis and ART status of patients**

Characteristics	Positive	Negative	p value
**Total (%)**	70 (38)	114 (62)	
**Age group (yrs)**			p < 0.05
5-12 (%)	4 (66.7)	2 (33.3)	
13-19 (%)	4 (66.7)	2 (33.3)	
20-27 (%)	2 (33.3)	4 (66.7)	
28-39 (%)	32 (30.8)	72 (69.2)	
40-49 (%)	16 (47.1)	18 (52.9)	
50-59 (%)	12 (42.9)	16 (57.1)	
	70	114	
**Gender**			p > 0.05
Male	28 (48.3)	30 (51. 7)	
Female	42 (33.3)	84 (66.7)	
**History of recent occurrence of respiratory tract infections**			p < 0.05
Yes	16 (38.1)	26 (61. 9)	
No	54 (38)	88 (62)	
**Conjunctivitis**			p < 0.05.
Yes	6 (33.3)	12 (66.7)	
No	64 (38.3)	102 (61.4)	
**Art status**			p < 0.05
ART	60 (37.5)	98 (62.5)	
Non ART	10 (38.5)	16 (61.5)	

The prevalence of Adenovirus infection in the HIV patients with respect to their gender was noticed to be higher in males than females, with males accounting for 49%, while the females 33%. There was no significant difference between the gender of HIV patients and Adenovirus co-infections (p > 0.05) (Table 1).

**Figure 1:**
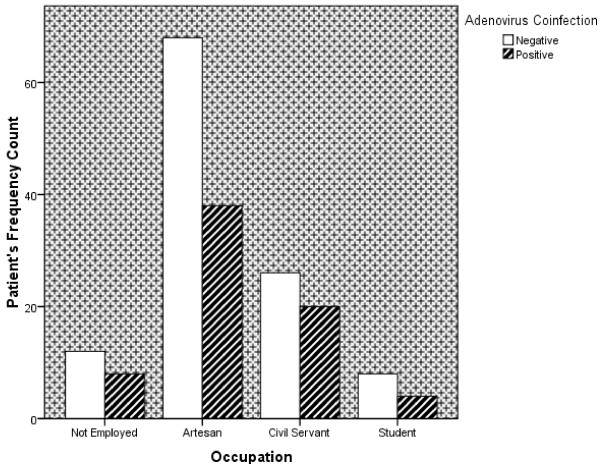
**Adenovirus infection prevalence rate in HIV patients by occupational grouping.** The p-value showed that there was significant difference between the Occupational grouping of HIV patients and Adenovirus co-infections (p < 0.05).

Results also showed that the total number of HIV patients employed which tested positive to the Adenovirus co-infections was 58 (31.5%) consisting of 38 (20.7%) Artisans and 20(10.8%) Civil Servants. Eight (8) HIV patients were unemployed having the Adenovirus co-infection, while four (4) HIV patients were students having the Adenovirus co-infections (Figure 1). The findings showed that there was significant difference between the occupational grouping of HIV patients and Adenovirus co-infections (p < 0.05).

**Figure 2:**
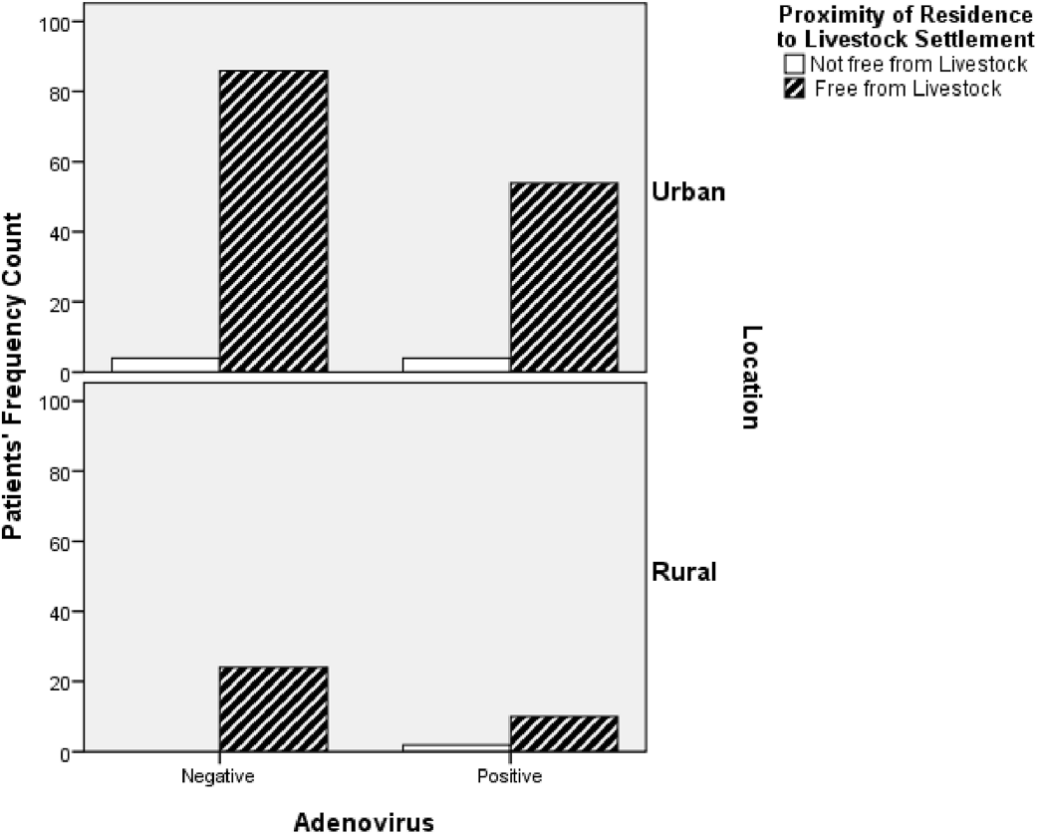
**The prevalence of adenovirus co-infection in the HIV patients categorized by their locations and proximity of residence to livestock settlement.** The p-value showed that there was significant difference between the locations and the proximity of the HIV Patients’ residence to Livestock Settlement with Adenovirus co-infection (p < 0.05).

The location of residence of patients was classified majorly into two groups; Urban and Rural settlements. The prevalence rate recorded in the urban settlers was slightly higher (39%) than what was recorded among the rural settlers (33%) (Figure 2). The p-value showed that there was significant difference between the locations of residence in HIV patients for both urban and rural dwellers, with Adenovirus co-infections (p < 0.05).

Prevalence rate of Adenovirus infection in HIV patients in relation to history of recent occurrence of respiratory tract infections (considering common symptoms such as cough, catarrh, sore throat and sneezing) with conjunctivitis noticed within 1-2weeks before visit to the clinic revealed that 12.5% of the total number of Adenovirus positive HIV patients had recent occurrences of respiratory tract infections and conjunctivitis before their visit to the clinic. However, 15% of all the Adenovirus negative HIV patients had the occurrence of both RTI and conjunctivitis (Table 1). The p-value revealed that there was significance difference between history of recent occurrence of Respiratory Tract Infections and Conjunctivitis with Adenovirus co-infections in HIV patients (p < 0.05).

**Figure 3:**
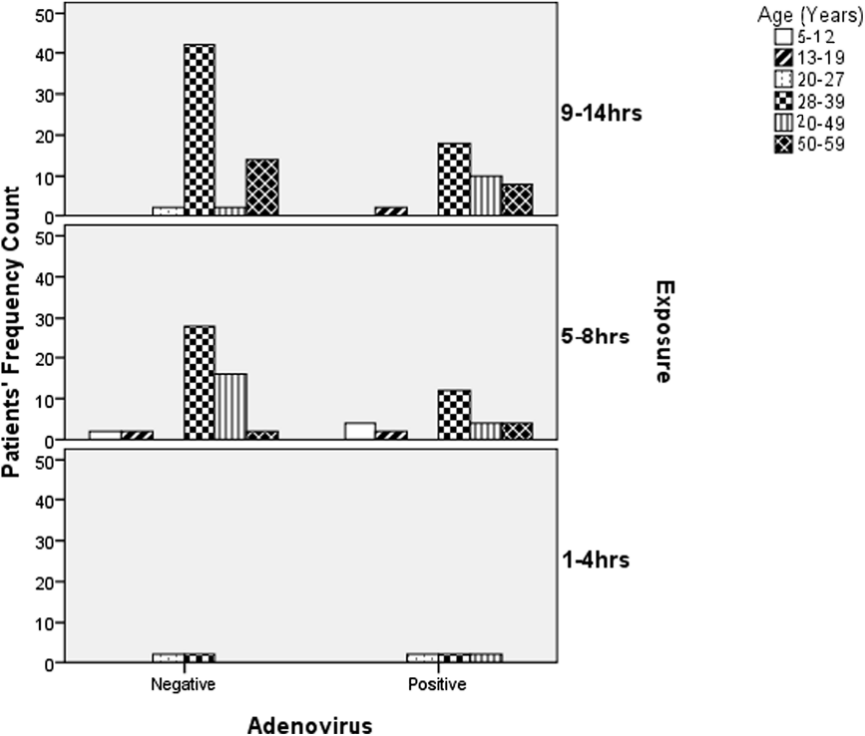
**Prevalence of adenovirus infections in HIV patients by age-matching with respect to the various categories of classification by patients’ exposure rate.** The p-value showed that there was no significant difference between the ages and Hours of daily exposure of HIV Patients with Adenovirus co-infection (p > 0.05).

The total number of patients with their residence close to livestock settlements was eight (8) of which 50% were positive to Adenovirus co-infections while the remaining 50% were negative (Figure 2). Also, classification was evaluated by correlating the average hours of exposure per day with the frequency of Adenovirus co-infection occurrence in the patients. The data obtained from this grouping was matched with age. The outcome for 5–8 hours of exposure group and 9–14 hours are represented in Figure 3. The class having the highest prevalence in both cases was the 28-39years age bracket. It accounted for 46.2% of cases across the age groups for the category of 5–8 hours exposure and 47.4% in the 9-14hours category. The p-values showed that there was no significant difference between the ages and Hours of daily exposure of HIV patients with Adenovirus co-infection for both 5–8 hours and 9–13 hours exposure (p > 0.05).

The percentage of the total population of ART patients positive to Adenovirus infection is 38%. Also, of all the 26 Non-ART patients, only 10 (38.5%) were positive to Adenovirus co-infections (Table 1). The p-value showed that there was significant difference between the ART status of HIV patients and Adenovirus co-infections (p < 0.05).

## Discussion

The Prevalence of Adenovirus co-infections in HIV patients in relation to age revealed a high prevalence rate among children within the school age (5–12 years). Children are at high risk of infection due to their frequent and consistent contact with carriers of the Adenoviral pathogen, their immune status which has already been suppressed with the Human Immunodeficiency Virus, is usually not well developed to ward off the Adenovirus infection. This is in line with the work of [21] which reported that ‘Adenovirus infection typically affects children from infancy to school age, but children of any age may be affected, including neonates. Young adults (13–19 years) in any setting of close quarters and stress may be affected. A study on Adenovirus involvement in diarrhoea in Children from the Northwestern part of Nigeria by [22] reported a relatively high prevalence rate for the same age group.

The gender classification in the prevalence study of Adenovirus infection in HIV patients was noted to be higher in males than it occurred in females. This is in tandem with the work of [21] submitting therein that Adenovirus urinary tract infections are more common in males. The prevalence of other syndromes does not appear to be affected by the sex of the individual. In their work, Aminu and Colleagues recorded a higher prevalence of enteric adenovirus infection in males than in females children-patients [22]. The high prevalence noticed in the male gender could be largely dependent on the behavioural disposition of male patients to orthodox medicine and unwillingness to consult healthcare facilities for medical attention. One major problem associated with the provision of healthcare services in Nigeria and some parts of Africa as portrayed by [23] was lack of education.

Considering the occupation of patients, the highest prevalence of Adenovirus was noticed among the Artesans and Civil Servants, with the Artesan group having the overall highest prevalence, except in the age group 40–49 where the prevalence in civil servants was higher than that noticed in the Artesans. This could be as a result of involvement in tasks that are associated with high rate of exposure. In 2007, a United States Air force training facility reported a cluster of severe respiratory tract illnesses associated with Ad14, which was tagged “a rare Human Adenovirus serotype”, it caused a protracted outbreak of respiratory illness among military recruits [24]. In a report from Zealand on Municipal Sewage Treatment (MST), where farms were said to be close to MST sites, farmers may most likely be at high risk of viral infections in cases of epidemic outbreak. Most often, people in the 28–39 and 40–49 years in this region are prone to occupational hazards with high risk of contracting the infection [25]. However, an observed low prevalence of Adenovirus infection in HIV patients who are students is attributed to the fact that in this part of the world majority are of school aged and often adequately monitored to ensure that they are in their classrooms as and when due. They are therefore less prone to daily tasks that are associated with high rate of exposure.

Location of residence revealed a slightly higher prevalence (39%) of the adenovirus infection in the patients who are urban settlers as opposed to what was noticed in patients from the rural areas (33%). This could be as a result of exposure to variety of activities in the urban areas which majorly are social, industrial and environmental in nature. A research conducted in New Zealand [25] reported that Human Adenovirus (HAdV) species C and F, and Norovirus GI and GII were frequently detected in municipal sewage and biosolid samples. It was further stated that viruses targeted by the research study - virus tool box (VTB) were detected in high concentrations in municipal raw sewage, biosolids, abattoir wastes, faecally contaminated environmental samples and also in human and animal faecal specimens. It was also pointed out that in New Zealand, many abattoirs discharge into municipal sewage treatment plants, and almost all of these plants are in close proximity to farming activities. Economic factors could also result to the high prevalence in the urban settlers as a result of Rural–urban migration, and hence leading to overcrowding in these cities.

History of recent occurrence of respiratory tract infections/conjunctivitis as a risk factor to Adenovirus co-infection, revealed that the population of patients with RTI’s alone far outweighed the population of patients with RTI and Conjunctivitis. There was however, a logical correlation between the occurrence of RTI/Conjunctivitis and Adenovirus co-infections with HIV in the patients (12.5%). A case report in Eket Coastal town [26], revealed a link between a history of antecedent upper respiratory tract infection or close contact with a “red eye” and adenovirus.

The possible risk factors as enumerated in the result included the closeness of patient’s residence to livestock settlement, which showed a prevalence rate of 50% for each group of patients which does not indicate any tangible relatedness to the prevalence of adenovirus infection in patients. However, this also does not bring about any indication towards zoonotic transmission of Adenovirus to humans, thereby suggesting host specificity in Adenovirus genera. Also, the average hours of patient’s daily exposure to infectivity showed highest prevalence in the age bracket 28–39 years for both 5–8 hours and 9–14 hours of exposure groups. This could be due to the social, occupational and behavioural involvements in day-to-day activities in this group of people. This is in consonance with the findings of Howieson and Hogan in 2005 on early winter deaths in Scotland [27].

Patients on Antiretroviral Therapy were found to have a slightly lower prevalence of Adenovirus infection compared to the Non - ART patients. The difference could be said to be negligible. It has been reported elsewhere that the demonstration of massive, rapid and largely irreversible HIV-mediated destruction of memory CD4+ T Cells predominantly occurring in the gut is suggestive that primary HIV infection maybe the only time that intervention could confer lasting immunological benefit [28] However, there was significant difference between the ART status of HIV patients and Adenovirus co-infections (p < 0.05). This supports the work done by Ghez *et al*., in 2000, where after 2 weeks of previous cycle of administering chemotherapy to an HIV patient for Burkitt lymphoma, Adenovirus-associated hemorrhagic cystitis was reported in the patient [29]. Nevertheless, vaccination still staggeringly stands out as a major way of preventing Adenovirus co-infections in human populations, especially in imunocompromised patients such as the HIV/AIDS patients.

There are several limitations regarding this study. Specific IgM antibodies have not been determined, hence discrimination between past or present infection cannot be ascertained. Only a subset of potential socioeconomic risk factors as well as other potential environmental risk factors were evaluated. The CD4 counts of the patients were not evaluated since subject recruited were those with confirmed HIV status based on their clinical records. The clinical manifestations of the co-infected patients was not elaborated. Also, the study did not differentiate and compare the prevalence of adenovirus in HIV patients with those with disease progression to AIDS. However, these limitations are being addressed in an on going study in our group.

## Conclusion

In summary, the prevalence rate of Adenovirus Respiratory tract infection in HIV patients obtained from this research is steadily alarming and higher than in most of the reported studies in Nigeria and some parts of Africa. Although, two cases of previous work in Nigeria, were conducted on enteric adenovirus among Children in Lagos and in the Northern Nigeria. Another was a case report in Eket, Nigeria on clinical features of Epidemic Adenovirus Ocular infection. Adenovirus infections and co-infections in immunocompromised patients could be aggravated by synergistic effects of the various factors examined in this research work, hence the reduction in the workforce of this society and also low output of infected individuals in this group may be inevitable if urgent response is not directed towards the disease control.

Children within the school age should be adequately taken care of in regions of high prevalence especially in arid and semi-arid regions. Children with symptomatic manifestations suggestive of adenovirus infection should be separated in day-care centres, and taken care of till they are considered fit to resume normal school activities.

There are possibilities of health workers acquiring nosocomial infections; therefore there is need for the use of protective coverings such as gloves, nose masks and laboratory/ward coats. Sterilization and other standard laboratory and operational practices essential in the day-to-day running of the laboratory and hospitals, should not be neglected and underrated. The development of vaccines against Adenovirus holds a promise to prevention of Adenovirus infection in individuals with high risk of infectivity, such as the Artesans as revealed in this study. Some Public workers (like military and paramilitary personnel) whose occupation require tedious engagement in activities conducted under harsh environmental conditions, should however be vaccinated during recruitment.

Governments and International Organisations should make adequate provisions in terms of funding and support for research into the gene therapy which is focused at modification of the Adenoviral fibre protein in targeting different cell lines, and hence its application in the treatment of diseases.

## Electronic supplementary material

Additional file 1: **Questionnaire for a Research Study on HIV-Adenovirus Respiratory Tract co-infection.** (DOC 30 KB)
