# Supplementary material for: Prevalence of adenovirus respiratory tract and hiv co-infections in patients attending the University of Ilorin, teaching hospital, Ilorin, Nigeria
Source: BMC Res Notes. 2014 Dec 3;7:870. doi: 10.1186/1756-0500-7-870 (PMC4289325; doi:10.1186/1756-0500-7-870)
Supplement: Supplementary file 1 — Additional file 1: Questionnaire for a Research Study on HIV-Adenovirus Respiratory Tract co-infection. (DOC 30 KB) [file 13104_2012_3384_MOESM1_ESM.doc]

**University of Ilorin Teaching Hospital**

**HIV/AIDS Antiretroviral Treatment (ART) Clinic Laboratory.**

**Questionnaire for a Research Study on HIV-Adenovirus Respiratory Tract co-infection.**

Please attempt all these questions with sincerity and to the best of your knowledge, in any case where there is no readily available answer or difficulty in answering any question, consult the Healthcare Personnel in charge. Please answer by ticking correctly the right option for each question. Your answers and any piece of information provided by you, would be treated with utmost confidentiality. Parents of patients are implored to help their children and wards to complete this form.

1. Age: 0-5 yrs ( ), 5-12 yrs ( ), 13-19 years ( ), 20-27 years ( ), 28-39years ( ), 40-49 years ( ), 40-49yrs ( ), 50-59yrs ( ), 60yrs and above ( )

50-59 years ( ), 60 years and above ( ).

2. Gender: M ( ) F ( )

3. Any Persistent Sore throat that has lasted more than a week? Yes ( ) No ( )

4. Any Persistent Sneezing in the past 2 weeks? Yes ( ) No ( )

5. Persisting Cough for more than 2 months?

Yes ( ) No ( )

6. Eye infection in the last 2-3 weeks (Conjunctivitis)?

Yes ( ) No ( )

7. Any regular/recent engagement in any long tedious hobby involving serious aerobics such as trekking or jogging in hazy or dusty environments?

   Yes ( ) No ( )

1. Any Medical history of Pneumonia in the last 3 years or more? Yes ( ) No ( )
2. Any Medical history of Tuberculosis? Yes ( ) No ( )
3. Any Medical history of whooping cough?   Yes ( ) No ( )
4. Any other Sexually Transmitted Disease (STD) previously diagnosed?                   Yes ( ) No ( )
5. If yes, kindly specify the name of the STD _______________________________
6. Any other respiratory tract disease diagnosed in the past? Yes ( ) No ( )
7. If yes, Specify the respiratory illness _______________________________
8. Location of Residence:                     Urban ( )   Rural ( )
9. Is your residence close to any livestock settlement? Yes ( ) No ( )
10. Occupation ____________________________
11. If Artesan, state the nature of work. ____________________________________
12. How well do you feed daily on the average?               Very well ( ) Well ( ) Averagely well ( ) Not so well ( ) Poorly ( )
13. How well do you take vitamin-containing foods such as fruits and vegetables? Very well ( ) Well ( ) Averagely well ( ) Not so well ( ) Poorly ( )
14. Have you ever been on any ART drugs?    Yes ( ) No ( )
15. If yes, what is your personal assessment on your adherence to taking your ART drugs in the past? Strict compliance ( ) Average compliance ( ) Weak compliance ( ) Poor compliance ( )
16. Are you presently on any ART drugs? Yes ( ) No ( )
17. If yes, what is your personal assessment on your adherence to taking your ART drugs at present? Strict compliance ( ) Average compliance ( ) Weak compliance ( ) Poor compliance ( )
18. Any vaccination against Adenovirus in the past before your present HIV status? Yes ( ) No ( )
19. Any vaccination against Adenovirus since the commencement of ART? Yes ( ) No ( )
20. How long on the average, do you stay outdoor daily? ____ Hours.
21. Any change of residence/environment in the last 3 weeks? Yes ( ) No ( )
22. In cases of change of residence, specify new residence _____________________.
23. In cases of change of environment, specify place/region visited ________________.
